# Unexpected effects of ivermectin and selamectin on inducible Cre^ER^ activity in mice

**DOI:** 10.1186/s42826-020-00069-7

**Published:** 2020-10-07

**Authors:** Peter A. Kropp, Gabrielle V. Rushing, Asa A. Brockman, Erin N. Z. Yu, Rebecca A. Ihrie, Maureen Gannon

**Affiliations:** 1 Department of Molecular Physiology and Biophysics, Vanderbilt University, Nashville, TN USA; 2 Present Address: Laboratory of Biochemistry and Genetics, National Institutes of Diabetes and Digestive and Kidney Diseases, National Institutes of Health, Bethesda, MD 20886 USA; 3 Neuroscience Program, Vanderbilt University, Nashville, TN USA; 4 Department of Cell and Developmental Biology, Vanderbilt University, Nashville, TN USA; 5 Department of Pathology, Immunology and Microbiology, Vanderbilt University Medical Center, Nashville, TN USA; 6 Department of Neurological Surgery, Vanderbilt University Medical Center, Nashville, TN USA; 7 Department of Medicine, Vanderbilt University Medical Center, Nashville, TN USA; 8 Department of Veteran’s Affairs, Tennessee Valley Health Authority, Nashville, TN USA

**Keywords:** Ivermectin, Selamectin, Transgenic mice, Cre^ER^

## Abstract

**Background:**

Anti-parasitics are frequently used in research animal facilities to treat a multitude of common infections, with pinworms and fur mites being amongst the most common. Ivermectin and selamectin are common oral and topical treatments for these infections, respectively. Although commonly thought to be innocuous to both the research animals and any transgenic elements that the animals may carry, evidence exists that ivermectin is capable of activating the recombinase activity of at least one Cre^ER^. The goal of the current study was to determine if there was an effect of either anti-parasitic agent on the activity of Cre^ER^ proteins in transgenic mice.

**Case presentation:**

We analyzed the offspring of transgenic mice exposed to either ivermectin or selamectin during pregnancy and nursing. Through analysis of reporter genes co-expressed with multiple, independently generated transgenic Cre^ER^ drivers, we report here that ivermectin and selamectin both alter recombinase activity and thus may have unintended consequences on gene inactivation studies in mice.

**Conclusions:**

Although the mechanisms by which ivermectin and selamectin affect Cre^ER^ activity in the offspring of treated dams remain unclear, the implications are important nonetheless. Treatment of pregnant transgenic mice with these anti-parasitics has the potential to alter transgene activity in the offspring. Special considerations should be made when planning treatment of transgenic mice with either of these pharmacologics.

## Background

Helminth infection is one of the most common types of parasitic infection in laboratory rodents. Although normally benign, these infections can eventually cause secondary effects including, but not limited to, heightening the host animal’s immune system. Such effects can not only confound studies of the immune system, but also alter the activity of any exogenous treatment or genetic element potentially recognized as foreign. Given these complications, in addition to the obvious problem of parasite burden, treatment of laboratory rodent colonies for removal of parasites is imperative for institutional veterinary care and research alike.

Amongst the most common treatments for helminthic parasitic infections are the pharmacological agents ivermectin and selamectin. Both drugs treat existing helminth infections by inducing paralysis in the parasites and thereby preventing normal life functions. Ivermectin is ingested orally whereas selamectin is administered topically. The commonly held belief is that ivermectin has minimal effect on mammalian systems and can thus be used without ill-effect on rodent colonies during ongoing pre-clinical studies. While this understanding of the safety of ivermectin appears to hold true in most circumstances, there is evidence that ivermectin treatment can have unintended effects on certain transgenic elements [4]. Variants of Cre recombinase, an enzyme used to conditionally inactivate genes, that are fused to a mutated form of the estrogen receptor (ER) and allow for temporal control of gene inactivation seem to be most sensitive to these treatments. Known as a Cre^ER^, this fusion protein is activated upon the administration of tamoxifen, an estrogen analog, resulting in translocation of the Cre^ER^ protein to the nucleus where it mediates DNA recombination at specific sequences known as *loxP* sites introduced into the desired locus.

A previous study has shown that ivermectin treatment in mice is capable of constitutively activating a Cre^ER^ specifically expressed in T-lymphocytes [4]. Given the concern this raises for temporal and cell type-specific regulation of gene inactivation, we performed independent pilot studies to examine the potential effects of ivermectin on other Cre^ER^ transgenes prior to a facility-wide treatment at our institution. Here we report the paradoxical findings that ivermectin and selamectin treatments could either aberrantly activate or prevent the activity of three independently generated transgenic Cre^ER^ fusion proteins.

## Case presentation

First, we examined the activity of an unpublished transgenic Elastase1-Cre^ER^ (Ela1-Cre^ER^) expressed in pancreatic acinar cells where the *elastase1* promoter drives expression of the *Cre*^*ER*^ (gift from Dr. Steve Konieczny, Purdue University). This transgene is expressed at such a high level, due to the strength of the promoter, that it has constitutive activity in approximately 50% of pancreatic acinar cells as evidenced by activation of a *lox-stop-lox-yellow fluorescent protein* (*lsl-YFP*) reporter construct knocked into the ubiquitously-expressed *Rosa26* locus [9]. In this construct, the *stop* sequence prevents transcription of the *YFP* mRNA. Activity of Cre recombinase removes the *stop* sequence thereby allowing transcription and translation of YFP and detection by live imaging or immunofluorescence. As noted above, the constitutive activity of the Ela1-Cre^ER^ normally results in YFP protein expression and detection in ~ 50% of pancreatic acinar cells even in the absence of tamoxifen.

**Fig. 1:**
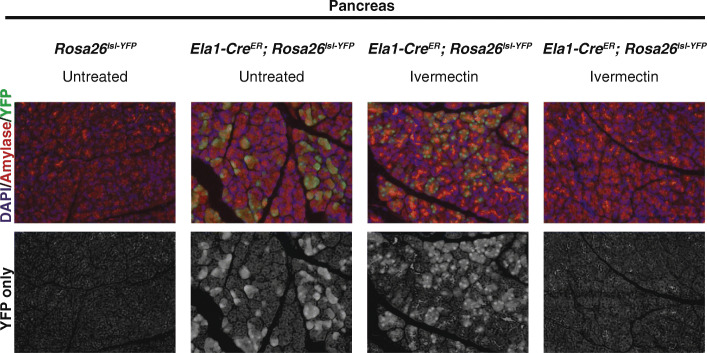
Ivermectin-induced silencing of *Ela1-Cre*^*ER*^ in the pancreas. Representative immunofluorescent images of postnatal day 21 pancreata. Sections are labeled with antibodies against amylase (red) for acinar cells and YFP (green) for expression of the *Rosa26*^*lsl-YFP*^ reporter of Cre activity. Colabeled with DAPI (blue, faint) for nuclei. Genotype and treatment group are indicated above images. No groups were administered tamoxifen at any time. Column 1 shows no YFP expression in untreated mice lacking the *Ela1-Cre*^*ER*^ transgene (3/3 mice analyzed). Column 2 shows constitutive Ela1-Cre^ER^ activity in untreated mice (7/7 double transgenic mice analyzed). Column 3 shows constitutive Ela1-Cre^ER^ activity in spite of ivermectin treatment (2/6 double trangenic mice analyzed) whereas column 4 shows lost constitutive Ela1-Cre^ER^ activity following ivermectin treatment (4/6 double transgenic mice analyzed)

For the purposes of this experiment, female mice homozygous for the *lsl*-*YFP* were mated to male mice carrying the *Ela1-Cre*^*ER*^. Following mating, female mice were placed on either standard chow diet (Lab Diet 5L0D, PMI Nutrition) or the diet containing ivermectin (Lab Diet 5L0D 12 ppm ivermectin, PMI Nutrition). Parental mice were maintained on the appropriate diet throughout pregnancy, birth of the pups, and until weaning at 21 days. At weaning, the pups were sacrificed, pancreata were harvested, and tissue sections were examined via immunofluorescent labeling for YFP (as in [6]). Unfortunately, one of the dams carrying the *Ela1-Cre*^*ER*^ transgene ate her pups, so we were limited to examining one litter of mice exposed to ivermectin. In this litter, 3/9 mice did not carry the *Ela1-Cre*^*ER*^ transgene and 6/9 mice carried both the *Ela1-Cre*^*ER*^ transgene and the *lsl-YFP* reporter (double transgenic). Normally, all six of these mice were expected to have detectable YFP expression. Indeed, all mice with both the transgene and reporter that were raised on the standard chow diet had detectable reporter expression (7/7 analyzed). Of the six double transgenic mice raised on ivermectin diet, 2/6 maintaind YFP expression. However, 4/6 double-transgenic mice raised on the ivermectin diet had no detectable YFP expression (Fig. 1) indicating that, by an unknown mechanism, expression, or activity, of the Ela1-Cre^ER^ is reduced or absent. The discrepancy between these findings and the findings of Corbo-Rodgers et al cannot be reconciled at this point since no mechanism of interaction between ivermectin and Cre^ER^ proteins has been identified.

The second experiment analyzed the activity of two Cre^ER^ transgenes expressed in the brain that label distinct neural stem cell populations within the ventricular-subventricular zone (V-SVZ). Empty spiracles homeobox 1 (Emx1-Cre^ER^) [5] is expressed in the developing cerebral cortex and in stem cells of the dorsal V-SVZ while GLI family zinc finger 1 (Gli1-Cre^ER^) [1] is expressed predominantly in the ventral forebrain, including the ventral V-SVZ. This experiment utilized a similar reporter to the one detailed above, but the fluorophore was a tdTomato following the *lsl* sequence in the *Rosa26* locus. This specific reporter is known as Ai14 [7]. Gli1-Cre^ER^; Ai14 and Emx1-Cre^ER^; Ai14 lines were either untreated, received an ivermectin diet (as above), or a topical selamectin treatment (Revolution, 0.3 mg, Zoetis, Inc) with fenbendazole diet (Lab Diet 5L0D, 150 ppm fenbendazole). Our normal experience with these lines is that no reporter gene expression is observed in the absence of tamoxifen administration. Ivermectin or selamectin treatment began at the start of gestation and continued in the postnatal period until animals were sacrificed at postnatal day 7 at which time brain slices were prepared for imaging. For all breeding pairs, Cre^ER^-negative females were paired with Cre^ER^-positive males. At no point were animals exposed to tamoxifen.

**Fig. 2:**
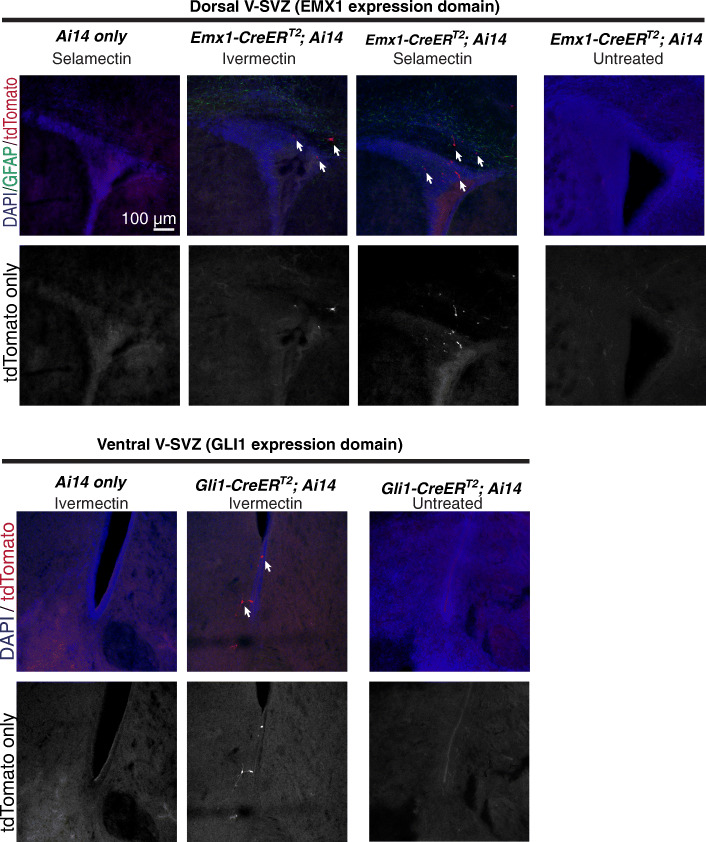
Ivermectin and/or selamectin-induced activation of *Emx1-Cre*^*ER*^ and *Gli1-Cre*^*ER*^ in the V-SVZ. Representative immunofluorescent images of postnatal day 7 dorsal (above) and ventral (below) V-SVZ. Sections are labeled with antibodies against GFAP (green) for glia/stem cells and tdTomato (red) for for expression of the Ai14 reporter of Cre activity. Colabeled with DAPI (blue) for nuclei. Region, genotype, and treatment group are indicated above images. No groups were administered tamoxifen at anytime. Tissue autofluoresence is visible as an artifact of imaging in control animals. Select tdTomato+ cells are indicated with arrows

From the Emx1-Cre^ER^; Ai14 line, we obtained two pups carrying both the Cre^ER^ and the *lsl-tdTomato* reporter that were exposed to ivermectin and one pup that was exposed to selamectin. From the Gli1-Cre^ER^; Ai14 line, we only obtained one pup carrying both the Cre^ER^ and reporter transgenes that was exposed to ivermectin. Immunofluorescent labeling showed aberrant tdTomato reporter expression in the absence of tamoxifen in both the dorsal (top) portion of the niche and within the corpus callosum of Emx1-Cre^ER^; Ai14 mice treated with either an ivermectin diet or topical selamectin (Fig. 2). In the Gli1-Cre^ER^; Ai14 pup, the ventral (bottom) subregion of the niche displayed aberrant tdTomato expression in the absence of tamoxifen treatment in the presence of ivermectin (Fig. 2). In all cases, unexpected tdTomato-positive cells were located within regions that would normally be targeted by the relevant transgenic Cre^ER^ line. Notably, these findings are consistent with those of Corbo-Rodgers et al in that ivermectin, and in this additional case selamectin, treatment can aberrantly induce Cre^ER^ activity in the absence of tamoxifen administration.

## Discussion and conclusions

Although these studies are limited in their scope, they both indicate that the highly-related antihelmitic drugs ivermectin and selamectin can alter the tamoxifen-independent activity of Cre^ER^ fusion proteins in the offspring of treated dams. In the case of Ela1-Cre^ER^, the expected constitutive activity was reduced or abolished. In the case of both Emx1-Cre^ER^ and Gli1-Cre^ER^, recombinase activity was abberantly activated in the absence of tamoxifen. Although the changes in activity of the respective Cre^ER^ lines were different between these studies, the results are concerning nonetheless.

The mechanism by which maternal ivermectin or selamectin affects activity of Cre^ER^ recombinases in offspring is not currently understood. The only other report of a similar finding [4] did not propose a mechanism and was instead more focused on the persistent activation of the Cre^ER^ for many weeks after cessation of ivermectin treatment. We speculate that ivermectin or selamectin treatment results in the generation of a metabolite capable of transmission transplacentally or through maternal milk thereby activating specific Cre^ER^ recombinases in the offspring. Ivermectin is known to be metabolized to no less than 10 different metabolites in the liver [10] and is capable of transmission via lactation [3]. It is thus possible that one of these metabolites is capable of binding to and activating the modified estrogen receptor used in Cre^ER^ transgenes. Further work into the scope of ivermectin and selamectin-derived metabolites, and their cross reactivity, would be enlightening.

The observed reduction in Ela1-Cre^ER^ activity requires a different explanation from that of the previous examples. In order to speculate about the mechanism of reduced activity, it is important to understand the nature of the constitutive activity to begin with. We hypothesize that the *elastase* promoter drives high enough levels of expression of the *Ela1-Cre*^*ER*^ transgene in pancreatic acinar cells to stochastically overcome the cytoplasmic retention of of the Cre^ER^ protein by heat shock proteins that is normally relieved by tamoxifen administration. Thus, abundance of Cre^ER^ protein drives translocation into the nucleus in accordance with mass action. This hypothesis is supported by evidence that the same *Cre*^*ER*^ sequence driven by a *Mist1* promoter has lower expression and no such constitutive activity [8] and personal communication from Dr. Koneiczny). While multiple possible mechanisms could exist to reduce transgene activity, the simplest explanation for our observations is silencing of the *Ela1-Cre*^*ER*^ transgene as a consequence of maternal ivermectin treatment. Such silencing of transgenic elements occurs at the epigenetic level with packaging of the transgenic element into heterochromatin [2]. Anecdotally, it is thought that immune activation in the host animal leads to recognition of the transgenic elements as “foreign” and thus the epigenetic silencing functions as a protective mechanism. Formal reports of such silencing rarely exist, but these events are well known amongst researchers using transgenic mice. In the case presented here, we hypothesize that a heightened immune response due to ivermectin treatment resulted in transgene silencing in approximately 2/3 of the offspring of treated dams. While we did attempt to measure Cre protein expression and localization with immunofluorescence, these experiments were unsuccessful at reliably labeling the Cre protein (data not shown). Importantly, Ela1-Cre^ER^ activity was reduced or absent in 4/9 mice born to a mother treated with ivermectin whereas no such reduction in activity was observed in untreated animals.

Regardless of the mechanisms by which ivermectin and selamectin affect Cre^ER^ recombinase or transgene expression, the studies presented herein highlight the importance of assessing transgenic lines following treatment with either of these agents. We hope that this information is useful to the field and invites special consideration should use of a Cre^ER^ coincide with a treatment regime of ivermectin or selamectin in other labs.

## Data Availability

Not applicable.

## Abbreviations

ER: Estrogen receptor
Ela1-Cre^ER^: Elastase1-Cre^ER^
lsl-YFP: lox-stop-lox-Yellow Fluorescent Protein
YFP: Yellow Fluorescent Protein
V-SVZ: Ventricular-Subventricular Zone
Emx1-Cre^ER^: Empty spiracles homeobox 1-Cre^ER^
Gli1-Cre^ER^: GLI family zinc finger 1-Cre^ER^
